# The Relationship Between Contraceptive Method Use and Return of Fecundity Among Women Attempting Pregnancy in Low- and Middle-Income Countries

**DOI:** 10.1215/00703370-10877719

**Published:** 2023-08-01

**Authors:** Alison Gemmill, Sarah E. K. Bradley, Blair O. Berger, Suzanne O. Bell

**Affiliations:** Department of Population, Family and Reproductive Health, Johns Hopkins Bloomberg School of Public Health, Baltimore, MD, USA; Public Health Demographer, Bethesda, MD, USA; Department of Population, Family and Reproductive Health, Johns Hopkins Bloomberg School of Public Health, Baltimore, MD, USA; Department of Population, Family and Reproductive Health, Johns Hopkins Bloomberg School of Public Health, Baltimore, MD, USA

**Keywords:** Fecundity, Low- and middle-income countries, Hormonal contraception, Fertility

## Abstract

One of the most common barriers to using effective family planning methods is the belief that hormonal contraceptives and contraceptive devices have adverse effects on future fertility. Recent evidence from high-income settings suggests that some hormonal contraceptive methods are associated with delays in return of fecundity, yet it is unclear if these findings generalize to low- and middle-income populations, especially in regions where the injectable is widely used and pressure to bear children is significant. Using reproductive calendar data pooled across 47 Demographic and Health Surveys, we find that the unadjusted 12-month probability of pregnancy for women attempting pregnancy after discontinuing traditional methods, condoms, the pill, and the IUD ranged from 86% to 91%. The 12-month probability was lowest among those who discontinued injectables and implants, with approximately 1 out of 5 women not becoming pregnant within one year after discontinuation. Results from multivariable analysis showed that compared with users of either periodic abstinence or withdrawal, users of the pill, IUD, injectable, and implant had lower fecundability following discontinuation, with the largest reductions occurring among women who used injectables and implants. These findings indicate that women’s concerns about potential short-term reductions in fecundity following contraceptive use are not unfounded.

## Introduction

Across diverse contexts, one of the most common barriers to using effective family planning methods is the belief that hormonal contraceptives and contraceptive devices have adverse effects on future fertility (Boivin et al. 2020; Payne et al. 2016; Williamson et al. 2009). In many regions of the world, especially where pressure to bear children is significant (Dyer 2007; Hollos et al. 2009), these barriers are pervasive and expressed by both men and women (Bornstein et al. 2020; Sedlander et al. 2018). Historically, these concerns have been dismissed as “misperceptions,” but emerging evidence indicates that such beliefs may in fact be rooted in personal experience or observations of others’ slower-than-expected returns of fecundity following contraceptive discontinuation (Bell et al. 2023).

Although previous reviews have generally concluded that one-year pregnancy rates following cessation of contraception are similar across a range of contraceptive types (Girum and Wasie 2018; Mansour et al. 2011), recent studies from high-income countries have indicated that some contraceptives might impact fecundity, especially in the short term. A 2020 study by Yland et al. (2020) using prospective cohort data collected in Denmark and North America found transient delays in return of fecundity among women who stopped use of oral contraceptives, the contraceptive ring, and some long-acting reversible contraceptives compared with barrier methods, with the largest decreases in fecundability among injectable and patch users. Importantly—and in contrast to prior studies—the authors employed a time-to-pregnancy study design for estimating fecundability, or the probability of conception per menstrual cycle, which is recommended to assess biologic fertility in a population (Joffe et al. 2005).

A key question is the extent to which the results from the Yland et al. (2020) study, which was conducted among individuals planning a pregnancy in Denmark and North America, generalize to women in low- and middle-income countries (LMICs) given several key differences in the contraceptive and fertility landscapes between high-income countries and LMICs. First, contraceptive formulations, which refer to the types of active ingredients and doses found in hormonal methods, are not uniform across settings (Sitruk-Ware et al. 2013). These formulations are linked with different mechanisms of action and rates of metabolization in the body that may influence the return of fertility following discontinuation. Second, there may be differences in the sociodemographic characteristics (e.g., age, parity) or life course stages associated with method preferences and use across settings. These context-specific differences in user profiles may limit the external validity of studies conducted in high-income countries.

Third, studies from high-income countries have mostly focused on patterns of fertility following oral contraceptive (pill) use (Barnhart and Schreiber 2009; Farrow et al. 2002), and the limited studies incorporating users of the contraceptive injectable or implant have been based on few study participants (Yland et al. 2020). This latter limitation is especially concerning given the rapidly increasing numbers of women in LMICs who use injectables and implants (Adetunji 2011; Anglewicz et al. 2019). Fourth, there are geographic differences in the burden of infertility, with higher prevalence of both primary and secondary infertility in LMICs than in high-income countries (Mascarenhas et al. 2012.) Reasons for these differences are not clear but may relate to differences in exposure to untreated reproductive tract infections (Larsen 2000), HIV infection (Gemmill et al. 2018), post-abortion complications, and injuries or infections caused or aggravated by childbirth.

To date, one study by Barden-O’Fallon and colleagues (2021) evaluated the return of fecundity among West and East African populations and found that the 12-month probability of pregnancy was lowest among those who had discontinued a hormonal method in order to become pregnant. The study, which used single-decrement life tables, was able to explore differences in these patterns by type of method discontinued, age, and parity but did not adjust for other known risk factors that might influence fecundability, such as socioeconomic status, partnership status, and health conditions and behaviors. This study also did not comprehensively describe potential short-term reductions in fecundity, which may be enough to dissuade women from using more effective methods (Barden-O’Fallon 2005).

The limited prior research on the topic of contraceptive use and return of fertility, as well as differing fertility contexts between the Global North and South, makes a compelling case for conducting a systematic evaluation in LMICs. While there are various ways to study fecundability in populations, the field of epidemiology has made great strides in investigating and identifying factors that impact individuals’ or couples’ ability to become pregnant using multivariable-adjusted time-to-pregnancy study designs (Joffe et al. 2005). This methodological approach, however, is rarely applied to populations from LMICs.

Using pooled, population-based data from 47 LMICs, the current study employs a retrospective time-to-pregnancy design to rigorously evaluate the return of fertility among women who discontinue contraception in order to become pregnant. Our multivariable approach accounts for differing distributions of risk factors for impaired fertility across populations that have not been fully considered by prior studies. This study, therefore, provides urgently needed quantitative evidence about method-specific impacts of use on return of fecundity in understudied settings. Ultimately, such information is of paramount importance to potentially validate and address—rather than dismiss and ignore—women’s concerns about contraception and to enhance person-centered counseling and contraceptive autonomy (Senderowicz 2020).

## Methods

### Data and Measures

We considered all Demographic and Health Surveys (DHS) conducted after 2010 that included a reproductive calendar module in which women were asked to provide reasons for discontinuing a method. If a country had more than one survey in this period, we used the most recent survey. Forty-eight DHSs conducted between 2010 and 2018 met the inclusion criteria; one survey (Yemen 2013) was excluded because information on an important covariate, education, was not included in publicly available data. Online appendix Table A1 displays a list of all 47 surveys and corresponding sample sizes included in our analysis.

DHS calendar data are retrospective month-by-month histories covering the five-year period prior to the interview. The calendars record women’s reproductive status in each month; possible states include pregnancy, birth, termination, and contraceptive use or nonuse. In any month when a woman reported discontinuing a contraceptive method, she was asked why she discontinued. We limited our study to women with a history of sexual activity who discontinued contraception because they “wanted to become pregnant” (*N* = 101,180 observations), which assumes that women in our study are exposed to the risk of pregnancy and are not taking deliberate action to avoid pregnancy.

Calendar data allowed us to determine the number of cycles (months) post–contraceptive discontinuation it took women to become pregnant or if they were unsuccessful during the period of observation. For all observations, time-to-pregnancy intervals began when women discontinued a method to become pregnant. Women were followed until one of the following endpoints, whichever occurred first: (1) a pregnancy occurred (based on self-report); (2) a woman began using contraceptives again after a period of nonuse and no observed pregnancy (censored); or (3) until three months prior to the interview (censored). This last endpoint avoids underestimating early pregnancies at the time of the interview that are underreported either because women do not yet recognize they are pregnant or because women do not yet want to disclose their pregnancy status. Women who may have been in the early stages of pregnancy at the time of the interview are still included in the study, but they are included as censored observations (i.e., in the population at risk of pregnancy until three months prior to the interview). Including all months up to the survey interview does not change the results. We also accounted for the presence of longer time-to-pregnancy intervals by censoring all observations at 12 months among those presumably at risk for pregnancy for more than a year.

**Fig. 1:**
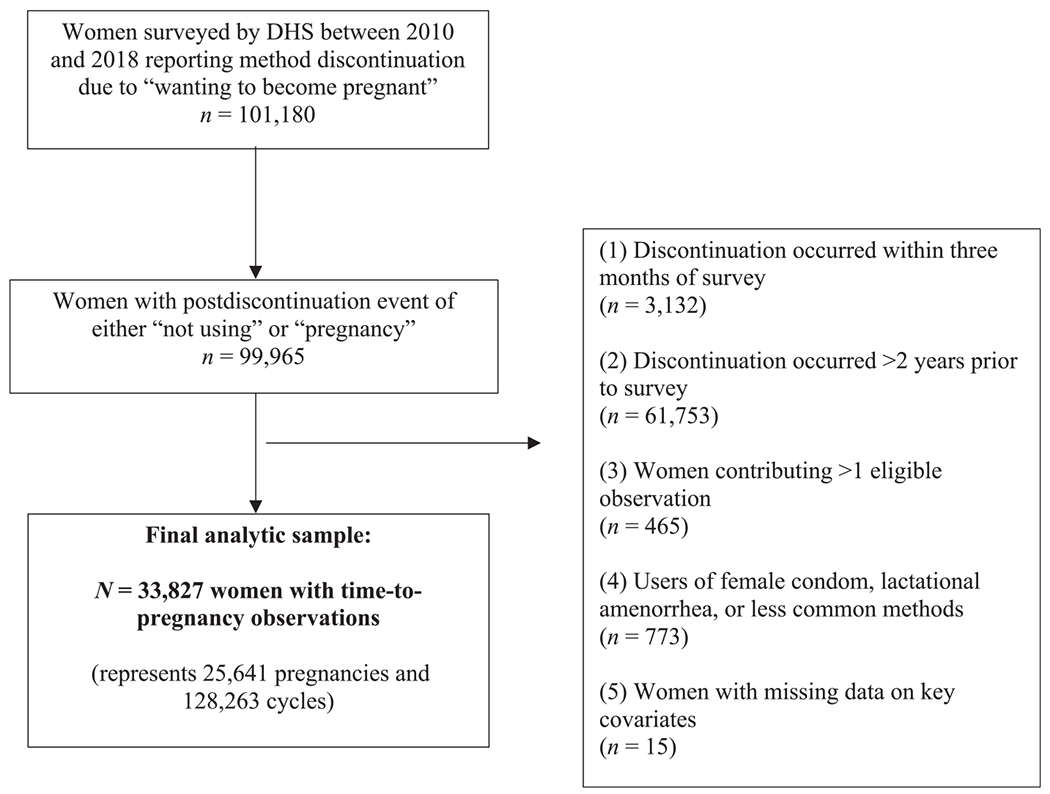
Flow diagram of study sample

We imposed several inclusion/exclusion criteria for our analytic sample (Figure 1). First, we restricted data to observations for which the month following contraceptive discontinuation was coded as either “not using” or “pregnancy” (*n* = 99,965 eligible observations). Second, we excluded observations for which contraceptive discontinuation occurred within the three months prior to the interview to account for potential underrecognition of pregnancies at the time of the survey (*n* = 3,132). Third, to reduce the threat of recall bias (Bradley et al. 2015), we limited our analysis to women who discontinued a contraceptive in the two years prior to the survey, which led to the exclusion of an additional 61,753 observations. In addition, if a woman contributed more than one eligible observation (*n* = 465 cases), we used the most recent one, so our unit of analysis is women, rather than episodes. We also excluded observations reporting less commonly used methods such as the female condom and those using the lactational amenorrhea method (*n* = 773). Lastly, we excluded those missing data on key covariates measured in all surveys (*n* = 15). The final sample size for our main analysis comprised 33,827 women attempting pregnancy, representing 25,641 pregnancies and 128,263 monthly cycles. Because the number of eligible women for analysis for some countries and methods was small (i.e., < 100 eligible cases), we pooled data across all surveys to ensure an adequate sample size for comparing time-to-pregnancy by prior contraceptive method used.

Our main independent variable—contraceptive method discontinued—was categorized by method type. We included methods in the analysis if at least 500 women in the pooled sample reported using that method to ensure an adequate number of method-specific observations for analysis; methods meeting this criterion are the oral contraceptive pill, IUD, injectable, male condom, implant, periodic abstinence, and withdrawal. For analysis, we grouped periodic abstinence and withdrawal into a category of traditional methods. The surveys included in our study did not collect further information on what type of pill, IUD, implant, or injectable was used, so we were unable to further disaggregate these methods by more specific characteristics (e.g., hormonal vs. copper IUD, different contraceptive formulations).

We considered several confounding factors for analysis that are probable risk factors for impaired fecundity or have been empirically associated with fecundability in prior studies. To account for reduced fecundability associated with age, we included a categorical variable with the following classification, which was based on respondents’ age at the time of discontinuation: 15–19, 20–29, 30–34, 35–39, and 40 or older.

Information on coital frequency and partner characteristics was unavailable. Instead, we used a three-category measure of union status that incorporates whether women were in a polygynous union (in a nonpolygynous union, in a polygynous union, and not in a union.)

Some research has suggested that infertility and fecundability are patterned by socioeconomic attributes such as education and income (e.g., Schrager et al. 2020). These patterns do not reflect inherent biological differences across socioeconomic position but instead are mediated by behavioral and lifestyle characteristics, as well as access to health care over the life course. We therefore included variables measuring socioeconomic position or access to health care that may help reduce the threat of residual confounding for risk factors correlated with impaired fecundity. The first, education, was coded as no education, primary, secondary, or higher. The second was a measure of household wealth that was coded according to the DHS wealth quintile classification for each country based on assets and household characteristics (i.e., poorest to richest; Rutstein and Johnson 2004). We also included a measure of urban versus rural residence based on urban and rural classifications for each country.

We included three sexual and reproductive health measures that may influence fecundability. Parity at the time of contraceptive discontinuation was assessed as a binary variable (nulliparous vs. parous). As noted earlier, exposure to untreated STIs may affect fecundity. We therefore included a measure of STI history that was assessed from questions asking if participants had an STI or symptoms of an STI (bad-smelling abnormal genital discharge or a genital sore or ulcer) in the 12 months prior to the survey; any indication of an STI or STI symptoms was coded as yes (vs. no indication). Our third measure assessed whether the respondent reported correct knowledge of the fertile period during an ovulatory cycle (yes or no), as this knowledge could be used to optimize the chance of pregnancy in each cycle (Capotosto 2021).

Our analyses also included two known risk factors for infertility, body mass index (BMI) and exposure to tobacco products (Rossi et al. 2014). We calculated BMI from weight and height data that were measured directly during the survey and categorized the measure according to the conventional WHO classification of adult underweight (< 18.5), normal (18.5–24.9), overweight (25.0–29.9), and obese (≥30.0). Our second measure was a composite binary indicator for use of tobacco products, determined from several questions assessing cigarette, cigar, and chewing tobacco use measured at the time of the survey (yes to using at least one product vs. none).

All covariates except for age and parity were measured at the time of the survey; age and parity status corresponded to when the woman discontinued contraception. All surveys in our analyses contained the following measures: age, parity, education, urban or rural residence, wealth, union status, and knowledge of the fertile period. Measures of BMI, recent history of an STI, and use of tobacco products were not available for all surveys. Therefore, in a sensitivity analysis, we tested whether our results were robust to a more extensive set of confounders in a subsample of countries that had all available covariates (*n* = 9,828).

### Statistical Analysis

First, we used the Kaplan–Meier method to estimate survival curves and one-year probabilities of pregnancy separately for each eligible contraceptive method. We also calculated median time to pregnancy for each method using the number of months when at least 50% of women became pregnant.

Second, we used Cox proportional hazard models for discrete survival data to model time to pregnancy and estimate fecundability ratios (FRs). FRs compare the odds of becoming pregnant between the exposed and unexposed groups; an FR less than 1 indicates that the exposed group (e.g., women discontinuing hormonal methods) experienced decreased odds of pregnancy compared with the unexposed or reference group (e.g., women discontinuing traditional methods) within the first year after contraceptive discontinuation. These models account for changes in the average fecundability of the population at risk over time, which result from more fecund women being removed from the risk set in later months. All models accounted for right-censoring and included country fixed effects to control for unobservable characteristics within each country. Tests of proportionality, including visual inspection of log-log survival plots, showed that the proportionality assumption was generally upheld.

We assumed that women using traditional methods or condoms served as appropriate counterfactuals for women using methods previously hypothesized to affect the return of fecundity following discontinuation, such as hormonal methods and IUDs. In the main analysis, women using traditional methods were selected as the reference category owing to concerns that condom users may differ from traditional method users with regard to their STI or HIV risk, which could impact time to pregnancy (Gemmill et al. 2018). That said, we also investigated whether inferences were the same when we used condom users as the reference group, as this would provide additional support for the idea that hormonal methods and IUDs influence future fecundity because of biological mechanisms of action.

We conducted several additional sensitivity analyses to evaluate the robustness of our findings. First, for users of injectables, we assumed an additional lag of three months to account for the possibility that women may have received their last injection in the month they reported discontinuing the method, and therefore could be fully protected from pregnancy up to three months. Second, as described earlier, we limited our sample to surveys that included the full set of covariates, including BMI and tobacco use, to examine whether our results were robust to their inclusion. Third, we conducted all analyses separately for women aged 40 or older, as any potential reductions in fecundity could be amplified for this age group. And finally, we expanded our sample to all eligible episodes within the entire five-year contraceptive calendar.

Following prior multicountry DHS studies (Bradley and Shiras 2022; Gemmill et al. 2018; Sarnak et al. 2023), we used custom weights accounting for complex sampling designs to allow each country to contribute equally to the pooled analysis; this approach ensures that results are not weighted more heavily toward surveys with larger sample sizes. Specifically, we multiplied the DHS-provided survey weights by a country-specific constant, such that the sample of women from each of the 47 countries in our analysis makes up 1/47th of the pooled sample, the derivation of which is outlined in detail elsewhere (Bradley and Shiras 2022). As an additional robustness check, we also conducted a jackknife analysis to ensure that results were not driven by countries with larger sample sizes. Statistics present unweighted *n*s and weighted percentages. Analyses were conducted in Stata 14.0 using the *svy* suite of commands. Ethics approval was obtained by the institutions that administered the surveys, and all analyses used anonymized databases.

## Results

Characteristics of the study sample are presented in Table 1. The majority of women were in their 20s (59%), had at least one prior birth (87%), and had at least a primary education (82%). Most women were in a union, and 8% reported being in a polygynous union. A little less than one third of women (29%) reported correct knowledge of the fertile period. Almost half (49%) of women in the weighted sample were from the sub-Saharan Africa region, whereas less than 10% were from either Europe or South Asia.

Descriptive statistics for users of each contraceptive method type are also presented in Table 1. Women who discontinued injectables and pills made up 31% and 26% of the weighted sample, respectively. Sixteen percent of the weighted sample discontinued either periodic abstinence or withdrawal (traditional methods), 13% discontinued condoms, 8% discontinued IUDs, and 6% discontinued implants. There were also sociodemographic and regional differences by type of contraceptive discontinued, which provide strong motivation for multivariable analysis.

**Fig 2.**
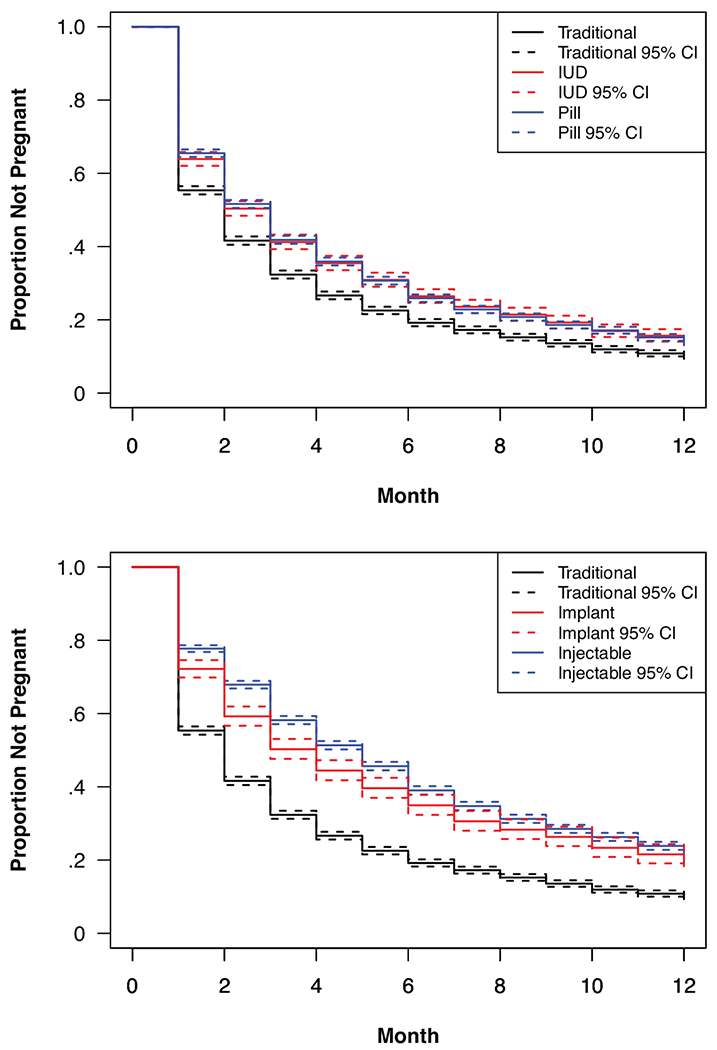
Kaplan–Meier survival curves of time-to-pregnancy by type of contraceptive method discontinued

Figure 2 presents Kaplan–Meier survival curves of time to pregnancy by type of method discontinued. For ease of comparison, both panels include the same reference curve for traditional methods (combined periodic abstinence and withdrawal) represented by the black line. The top panel presents additional curves for the IUD and the pill, and the bottom panel presents additional curves for the implant and the injectable. Condoms are not included because they overlap closely with traditional methods.

Both figures demonstrate that users of the IUD, pill, implant, and injectable experience longer times to pregnancy than users of traditional methods. These curves are quantified in Table 2, which displays the median time to pregnancy (TTP) and 12-month probabilities of pregnancy observed for each method. The median TTP for traditional method and condom users following discontinuation is two months, while median TTP for pill and IUD users is three months. Those using the implant and the injectable experience a median TTP of four and five months, respectively. The median TTP for users of the injectable shortens to two months after accounting for a three-month lag.

As evidenced by the Figure 2 curves and Table 2 data, there are also differences in 12-month probabilities of pregnancy. Traditional users had the highest probability at 91% (95% CI: 89.8, 91.4), followed by women using the condom (88%; 95% CI: 86.6, 88.6), the pill (87%; 95% CI: 86.0, 87.8), and the IUD (86%; 95% CI: 84.3, 87.7). Women discontinuing injectables and implants had the lowest 12-month probabilities of pregnancy—each at 80% (95% CI: 78.9, 81.0 and 77.3, 82.6, respectively). Thus, among women discontinuing injectables or implants in order to become pregnant, approximately 1 in 5 did not achieve pregnancy in a year, on average, compared with approximately 1 in 10 women using traditional methods.

Women aged 40 or older had longer median TTPs by contraceptive type discontinued, as well as reductions in the 12-month probability of pregnancy for all methods. Among older women who discontinued traditional methods, the 12-month probability of pregnancy was 81% (95% CI: 75.1, 86.1), which is approximately 10 percentage points lower than the probability among all women of reproductive age who also discontinue traditional methods; this difference likely captures well-known age-related declines in fecundity. Notably, 12-month probabilities of pregnancy were much lower for older women who discontinued either hormonal methods or the IUD compared with all women of reproductive age. For example, about 64% (95% CI: 56.9, 69.1) of women aged 40 or older became pregnant within a year following discontinuation of injectables, on average, compared with 80% (95% CI: 78.9, 81.0) among all women of reproductive age.

Table 3 presents results from a multivariable model that accounts for potential differences in underlying fecundity between women. The baseline model adjusts for age, parity, education, urban or rural residence, union status, education level, and knowledge of the fertile period; the model also includes country fixed effects. The first column in Table 3 employs users of traditional methods as the reference category. Compared with these individuals, users of the pill, IUD, injectable, and implant had lower fecundability ratios following contraceptive discontinuation. The largest reductions in odds occurred among women who used injectables or implants: 0.41 (95% CI: 0.38, 0.45) and 0.51 (95% CI: 0.45, 0.58), respectively. Patterns are largely similar when employing condom users as the reference group (column 3), although FRs increase slightly. There were no significant differences in fecundability between condom users and traditional users.

Findings remain similar after conducting several sensitivity analyses, with some exceptions. First, after accounting for a three-month lag for injectable users, we found that the adjusted FR (compared with traditional users) increases from 0.42 (95% CI: 0.38, 0.58) to 0.66 (95% CI: 0.60, 0.72) (not shown). Second, we reran our analyses among a subset of surveys that collected information on the full set of covariates (column 5 of Table 3) and found that results do not change substantially. Third, as shown in column 7, and mirroring our age-specific results in Table 2, we find large reductions in fecundability ratios for women aged 40 or over by contraceptive type after adjustment for covariates. Fourth, when we expand our analysis to all eligible episodes that occur within five years of the survey, we find similar results for all methods except for condom users. Specifically, condom users have a lower fecundability ratio than traditional users that was not observed in our main analysis (FR for condom users is 0.81; 95% CI: 0.76, 0.87). Finally, results do not change after conducting a jackknife analysis.

## Discussion

In this analysis using pooled data from 47 LMICs, we found that some contraceptive methods when used prior to attempting to get pregnant are associated with transient delays in return of fecundity, with the longest delays occurring among women who discontinued injectables and implants. These relationships persisted after adjustment for important confounders, suggesting that women’s concerns about potential short-term reductions in fecundity following use of certain contraceptives are not unfounded.

We acknowledge that our results can be interpreted differently by fertility researchers. While our findings show that at least half of women will become pregnant within 2–3 months following discontinuation of traditional methods, condoms, the pill, and the IUD, we see different patterns for injectables and implants—two methods that are widely promoted and used across LMICs. More importantly, because fecundity is heterogeneous (Leridon 2007), the median estimates of time to pregnancy presented in Table 2 do not sufficiently capture how the entire distribution of time to pregnancy shifts to the right following discontinuation of hormonal methods. This distributional shift leads to lower 12-month probabilities of pregnancy for users of hormonal methods than for those who discontinue traditional methods. These impacts are rarely discussed by family planning researchers but may lead to noticeable differences within communities and social networks (Sedlander et al. 2018). As an example, in a hypothetical population of 10,000 women who discontinue injectables or implants, nearly 2,000 of these women may still not experience pregnancy one year later. This delay is twice as long as what we would expect from a population of 10,000 women who discontinue traditional methods.

Our study corroborates some, but not all, findings from Yland et al. (2020), who evaluated the association between pregravid contraceptive use and subsequent fecundability in Denmark and the United States. Similar to Yland et al., we find that users of injectables have the longest delays in return of fertility; both studies found average or median times-to-pregnancy of about five months (although the range from Yland et al. extended to eight months). However, our study findings diverge from those of Yland et al. (2020) regarding other contraceptive types. For example, whereas Yland et al. (2020) found that users of IUDs had increased time to pregnancy compared with users of barrier methods, we do not find this association. Our study also differs in that we find substantial reductions in fecundability ratios among implant users, whereas this relationship was not apparent in the Yland et al. (2020) study. These differences could arise from use of different formulations of hormonal contraceptives across contexts as well as the larger number of implant observations in the current study: *n* = 1,373 in this study versus *n* = 186 in Yland et al. (2020).

Our study also builds on the findings of Barden-O’Fallon et al. (2021), which found lower returns to pregnancy by 12 months among women in West and East Africa who discontinued hormonal methods. Taken together, these results indicate that previous reviews on the topic (Girum and Wasie 2018; Mansour et al. 2011), which suggested no impact, should be urgently updated to incorporate new evidence. Moreover, future research should evaluate the potential biochemical or biobehavioral pathways underpinning these relationships, which so far remain speculative.

Critically, these findings have implications for family planning programs in LMICs. Several global efforts, including FP2030 and the Sustainable Development Goals, emphasize increasing the use of modern contraceptives. However, these efforts are potentially at odds with women’s contraceptive preferences and concerns. Our findings bolster the critical need for increased person-centeredness in family planning counseling and provision, in line with wider calls to shift the needle on family planning “successes” away from just “use” to maximizing autonomy and use of preferred methods (Senderowicz 2020). More concretely, our findings indicate that the acceptability of delayed return of fertility should be evaluated when recommending and choosing contraceptive methods.

Our study has several strengths. First, we use population-based data that allowed us to account for potential differences in population composition and underlying fecundity across settings. Second, our sample had a large number of observations of women who discontinued injectables and implants. By contrast, injectable and implant users from the Yland et al. study (2020) represented only 0.5% (*n* = 94) and 1.0% (*n* = 186) of participants, respectively. Third, there are few studies from LMICs that investigate determinants of fecundability and infertility. Our use of calendar data adds to the limited literature by employing a time-to-pregnancy study design most often used in higher resource settings.

There are also several limitations to note. First, except for age and parity, all covariates were measured at the time of the survey, not at the time of contraceptive discontinuation. It is unclear if this type of misclassification might bias our main results, since our measure of prior contraceptive type used does not suffer from this same error. Second, we relied on retrospective calendar data, which are subject to recall bias and other types of reporting errors (Bradley et al. 2015). In their report assessing quality of the DHS contraceptive calendar, for example, Bradley and colleagues’ (2015) results suggest worse reporting for events further in the past. To address this concern, we limited our observations to the two years prior to the survey, although sensitivity analyses using all five years prior to the survey generally yield similar results. Third, for users of injectables, we did not have data on when women received an injection relative to when she reported discontinuation, although we did include a three-month lag in our sensitivity analyses. Fourth, owing to data limitations, we could not distinguish between method type for injectables and IUDs. We note, however, that in low-resource settings, many IUDs are copper, rather than hormonal, and some injectables are formulated to provide contraception for one or two rather than three months (e.g., combined injectables and NET-EN/EV). While DMPA, which provides three months of protection, remains the most common type of injectable in LMICs, there is some variation in the injectable mix across settings (Laryea et al. 2016), although this is not well-documented.

Fifth, because interviewers could record only one contraceptive method per month, discontinuation of multiple contraceptive methods (i.e., dual use) is not possible to measure. Reports of using traditional methods like abstinence and withdrawal may also suffer from poor reliability compared with use of hormonal methods (Callahan and Becker 2012). Sixth, the DHS data we used do not include information on regular sexual activity, partners, and other measures that could influence fecundability. Measures of people’s underlying fecundity or propensity for infertility were also not possible to estimate. Seventh, we did not include measures of sexual violence and intimate partner violence in our study, even though prior research suggests that these experiences may influence health outcomes, including STI transmission (Barber et al. 2018; Campbell 2002; Coker, Sanderson et al. 2000; Coker, Smith et al. 2000).

A final limitation is that we cannot validate two key assumptions of this study: that women’s desire to become pregnant following contraceptive discontinuation was stable over time and that women were actively trying to become pregnant over the exposure period. As noted in prior research, short-term changes in pregnancy intention have been well-documented in several contexts (Sennott and Yeatman 2012; Trinitapoli and Yeatman 2018), and pregnancy ambivalence is also common (Sennott and Yeatman 2018; Tobey et al. 2020).

## Conclusion

Many women in LMICs either do not use contraception or discontinue contraceptive methods for fear that contraception will inhibit their future fertility. Although return of fecundity is acknowledged in the WHO Medical Eligibility Criteria for Contraceptive Use (MEC) (World Health Organization et al. 2018), contraceptive counseling protocols and tools used in LMICs may not include nuanced information about return to fecundity following discontinuation, even though this remains a common concern among women. Furthermore, although the WHO MEC discusses potential effects of injectables on return to fertility, there is no mention of other reversible methods. Our novel findings on the contraceptive implant, in particular, warrant increased attention within the family planning community. While we recognize that the present analysis has limitations, we hope our study prompts further research on this historically overlooked topic.

Ultimately, our results indicate that delayed return to fecundity after discontinuing some hormonal methods is a common experience in LMICs, providing what we believe to be some of the first multicountry evidence to validate women’s lived experiences from these regions. Contraceptive counseling policy and programs, therefore, should consider integrating this information to provide a fuller picture of the range of time-to-pregnancy experiences following contraceptive discontinuation, especially for injectables and implants. While information about potential declines in fecundity is just one criterion that may influence women’s contraceptive use, individuals have a right to this knowledge so that they can make informed choices (Senderowicz 2020).

## Supplementary Material

Appendix

## Figures and Tables

**Table 1:** Characteristics of entire study sample and by contraceptive method discontinued (*n*s, with percentages in parentheses)

	Entire Sample (*n* = 33,827)	Traditional Methods^a^ (*n* = 7,442)	Condom (*n* = 6,325)	Pill (*n* = 8,311)	Injectable (*n* = 7,875)	IUD (*n* = 2,501)	Implant (*n* = 1,373)
Age at Discontinuation							
<20	2,697 (7.2)	634 (6.7)	656 (11.5)	648 (6.4)	641 (7.9)	48 (1.4)	70 (6.7)
20–29	21,114 (58.5)	4,701 (57.4)	4,355 (60.4)	5,216 (60.0)	4,521 (57.3)	1,512 (56.8)	809 (60.3)
30–34	6,560 (21.9)	1,347 (22.4)	944 (18.2)	1,600 (21.7)	1,717 (22.2)	642 (27.2)	310 (19.9)
35–39	2,305 (8.2)	502 (8.7)	256 (7.0)	551 (7.4)	665 (8.5)	204 (9.8)	127 (8.8)
40–49	1,151 (4.2)	258 (4.8)	114 (3.0)	296 (4.5)	331 (4.0)	95 (4.8)	57 (4.1)
Prior Births at Discontinuation							
No	4,666 (12.7)	1,351 (17.6)	1,715 (30.3)	943 (12.3)	574 (8.0)	20 (0.3)	63 (5.1)
Yes	29,161 (87.3)	6,091 (82.4)	4,610 (69.7)	7,368 (87.7)	7,301 (92.0)	2,481 (99.7)	1,310 (94.9)
Education							
None	6,240 (18.3)	1,419 (10.7)	799 (8.5)	1,756 (21.3)	1,634 (24.3)	289 (10.9)	343 (28.3)
Primary	8,466 (28.8)	1,633 (26.2)	917 (16.3)	1,962 (27.2)	3,235 (40.8)	276 (11.2)	443 (33.5)
Secondary	14,104 (37.5)	3,130 (38.8)	3,064 (47.0)	3,635 (39.4)	2,554 (29.8)	1,282 (48.3)	439 (28.7)
Higher	5,017 (15.4)	1,260 (24.3)	1,545 (28.2)	958 (12.2)	452 (5.1)	654 (29.6)	148 (9.5)
Residence							
Rural	20,006 (55.0)	4,489 (43.8)	3,376 (38.7)	4,895 (55.8)	5,206 (67.7)	1,227 (48.3)	813 (61.5)
Urban	13,821 (45.0)	2,953 (56.2)	2,949 (61.3)	3,416 (44.2)	2,669 (32.3)	1,274 (51.7)	560 (38.5)
Wealth Quintile							
Poorest	5,629 (15.0)	1,489 (16.8)	581 (8.6)	1,396 (14.8)	1,620 (17.9)	318 (12.6)	225 (13.2)
Poorer	6,716 (18.1)	1,681 (19.1)	916 (13.4)	1,754 (16.6)	1,684 (20.9)	433 (17.8)	248 (18.2)
Middle	6,627 (19.4)	1,420 (17.8)	1,275 (19.3)	1,613 (19.3)	1,604 (20.6)	472 (20.8)	243 (16.7)
Richer	6,965 (21.9)	1,348 (19.4)	1,530 (26.3)	1,708 (22.1)	1,543 (21.1)	571 (21.2)	265 (22.6)
Richest	7,890 (25.7)	1,504 (27.0)	2,023 (32.4)	1,840 (27.2)	1,424 (19.6)	707 (27.6)	392 (29.4)
Union Status							
In nonpolygynous union	30,827 (86.4)	7,075 (92.5)	5,859 (82.4)	7,566 (87.0)	6,789 (82.9)	2,427 (95.9)	1,111 (80.2)
In polygynous union	1,840 (8.2)	217 (4.7)	119 (4.0)	505 (9.0)	748 (11.3)	52 (2.9)	199 (16.0)
Not in union	1,160 (5.4)	150 (2.8)	347 (13.6)	240 (3.9)	338 (5.8)	22 (1.3)	63 (3.9)
Correct Knowledge of Fertile Period							
No	24,096 (71.0)	4,895 (60.0)	4,272 (65.0)	6,070 (73.6)	6,283 (79.2)	1,576 (61.5)	1,000 (73.9)
Yes	9,731 (29.0)	2,547 (40.0)	2,053 (35.0)	2,241 (26.5)	1,592 (20.8)	925 (38,5)	373 (26.1)
Region							
Sub-Saharan Africa	9,933 (49.0)	973 (24.3)	872 (37.4)	2,390 (51.5)	4,426 (68.9)	206 (11.7)	1,066 (84.1)
South Asia	10,904 (5.8)	3,869 (12.6)	4,087 (17.7)	2,186 (2.5)	227 (2.0)	530 (2.3)	5 (0.5)
North Africa/Middle East	4,180 (14.4)	573 (13.8)	246 (6.5)	1,666 (20.8)	432 (4.1)	1,237 (53.5)	26 (1.5)
Latin America and the Caribbean	3,761 (10.3)	1,005 (16.8)	678 (14.6)	806 (8.6)	1,014 (9.1)	158 (5.6)	100 (3.4)
East Asia	3,867 (11.3)	534 (10.1)	111 (2.5)	1,172 (14.1)	1,753 (15.4)	121 (4.5)	176 (10.6)
Europe	1,182 (9.2)	488 (22.4)	331 (21.4)	91 (2.5)	23 (0.6)	249 (22.5)	0 (0.0)

*Note:* Data represent unweighted *n*s and weighted percentages.

a Consists of periodic abstinence and withdrawal.

**Table 2:** Median time to pregnancy (TTP) and 12-month probability of pregnancy (95% CIs) by contraceptive method discontinued, for all women and for women aged 40 or older

	All Women			Women Aged 40 and Older		
Method	*n*	Median TTP	12-Month Probability of Pregnancy	*n*	Median TTP	12-Month Probability of Pregnancy
Traditional^a^	7,442	2	90.6 (89.8, 91.4)	258	2	81.4 (75.1, 86.1)
Condom	6,325	2	87.7 (86.6, 88.6)	114	3	75.2 (64.9, 82.5)
Pill	8,311	3	86.9 (86.0, 87.8)	296	3	74.6 (67.9, 79.9)
IUD	2,501	3	86.1 (84.3, 87.7)	95	9	62.8 (48.4, 73.1)
Injectable	7,875	5	80.0 (78.9, 81.0)	331	6	63.5 (56.9, 69.1)
Injectable (with three-month lag)	7,875	2	84.7 (83.6, 85.8)	331	4	68.6 (61.5, 74.4)
Implant	1,373	4	80.1 (77.3, 82.6)	57	8	58.2 (39.6, 71.0)

a Consists of periodic abstinence and withdrawal.

**Table 3:** Adjusted fecundability ratios (FR; 95% CIs) by contraceptive method discontinued

	Traditional Methods^a^ as Reference Group^b^		Condoms as Reference Group^b^		Adjusting for Full Set of Covariates^c^		Women Aged 40 or Older^b^	
Method	FR	95% CI	FR	95% CI	FR	95% CI	FR	95% CI
Traditional^a^	ref.	—	1.08	0.97, 1.20	ref.	—	ref.	—
Condom	0.92	0.83, 1.03	ref.	—	0.92	0.79, 1.08	0.79	0.41, 1.52
Pill	0.65	0.59, 0.71	0.70	0.63, 0.78	0.66	0.57, 0.76	0.48	0.30, 0.78
IUD	0.66	0.58, 0.75	0.71	0.62, 0.81	0.64	0.51, 0.80	0.25	0.12, 0.53
Injectable	0.41	0.38, 0.45	0.45	0.41, 0.50	0.42	0.37, 0.48	0.37	0.23, 0.59
Implant	0.51	0.45, 0.58	0.55	0.48, 0.63	0.56	0.47, 0.68	0.35	0.19, 0.65

a Consists of periodic abstinence and withdrawal.

b Models adjust for age, parity, education, residence, union status, and country fixed effects.

c Models adjust for age, parity, education, residence, union status, BMI, smoking, recent history of STIs, and country fixed effects.
