# Supplementary material for: The Relationship Between Contraceptive Method Use and Return of Fecundity Among Women Attempting Pregnancy in Low- and Middle-Income Countries
Source: Demography. Author manuscript; Available in PMC 2023 Sep 27. (PMC10529236; doi:10.1215/00703370-10877719)
Supplement: Appendix [file NIHMS1917394-supplement-Appendix.pdf]

Online Appendix for:

The Relationship Between Contraceptive Method Use and Return of Fecundity Among Women Attempting Pregnancy in Low- and Middle-Income Countries

Alison Gemmill, Sarah E.K. Bradley, Blair O. Berger, and Suzanne O. Bell

Table A1. List of DHS surveys and number of observations per survey.

Survey	Region	Number of observations
Afghanistan 2015	Middle East and North Africa	1,112
Albania 2017-18	Europe	165
Angola 2015-16	Sub-Saharan Africa	74
Armenia 2015-16	Europe	161
Benin 2017-18	Sub-Saharan Africa	461
Burkina Faso 2010	Sub-Saharan` Africa	414
Burundi 2016-17	Sub-Saharan Africa	413
Cambodia 2014	East Asia	723
Colombia 2015	Latin America and the Caribbean	663
Comoros 2012	Sub-Saharan Africa	62
Egypt 2014	Middle East and North Africa	1,940
Ethiopia 2016	Sub-Saharan Africa	690
Gambia 2013	Sub-Saharan Africa	78
Ghana 2014	Sub-Saharan Africa	224
Guatemala 2014-15	Latin America and the Caribbean	941
Guinea 2018	Sub-Saharan Africa	112
Honduras 2011-12	Latin America and the Caribbean	1,236
India 2015-16	South Asia	9,925
Indonesia 2017	East Asia	2,230
Jordan 2017-18	Middle East and North Africa	1,128
Kenya 2014	Sub-Saharan Africa	569
Kyrgyz Republic 2012	Europe	225
Lesotho 2014	Sub-Saharan Africa	195
Liberia 2013	Sub-Saharan Africa	109
Malawi 2015-16	Sub-Saharan Africa	831
Maldives 2016-17	South Asia	47
Mali 2018	Sub-Saharan Africa	233
Mozambique 2011	Sub-Saharan Africa	174
Myanmar 2015-16	East Asia	547
Namibia 2013	Sub-Saharan Africa	291
Nepal 2016	South Asia	265
Niger 2012	Sub-Saharan Africa	430
Nigeria 2018	Sub-Saharan Africa	669
Pakistan 2017-18	South Asia	667
Papua New Guinea 2016-18	East Asia	132
Peru 2012	Latin America and the Caribbean	921

Rwanda 2014-15	Sub-Saharan Africa	534
Senegal 2017	Sub-Saharan Africa	459
Sierra Leone 2013	Sub-Saharan Africa	254
South Africa 2016	Sub-Saharan Africa	107
Tajikistan 2017	Europe	101
Tanzania 2015-16	Sub-Saharan Africa	663
Timor-Leste 2016	East Asia	235
Turkey 2013	Europe	530
Uganda 2016	Sub-Saharan Africa	659
Zambia 2018	Sub-Saharan Africa	595
Zimbabwe 2015	Sub-Saharan Africa	633
**Total**		**33,827**
